# Cancer-like Hallmarks of Endometriosis: The Role of Estrogen Signaling and Stem Cell Plasticity

**DOI:** 10.3390/ijms27104510

**Published:** 2026-05-18

**Authors:** Pietro Giulio Signorile, Alfonso Baldi, Antonella Mazzotti, Manuela Montanaro, Mariarosaria Boccellino

**Affiliations:** 1 Italian Endometriosis Foundation, 00197 Rome, Italy; research@endometriosi.it (P.G.S.); a.baldi@unilink.it (A.B.); 2 Department of Life Science, Health and Health Professions, Link Campus University, 00165 Rome, Italy; m.montanaro@unilink.it; 3 Unit of Pathology, Monaldi Hospital, A.O. dei Colli, 80131 Naples, Italy; antonella.mazzotti@tiscali.it

**Keywords:** endometriosis, estrogen signaling, stem cell plasticity, cancer-like hallmarks, ovarian cancer, tumorigenesis, epigenetics, tumor microenvironment

## Abstract

Endometriosis is a chronic estrogen-dependent inflammatory disease affecting approximately 10% of women of reproductive age and characterized by ectopic endometrial-like tissue growth. Although traditionally considered a benign gynecological condition, increasing evidence indicates that endometriosis shares several molecular and cellular features with malignant processes, including enhanced proliferation, resistance to apoptosis, invasive behavior, and the ability to remodel the surrounding microenvironment. Recent studies suggest that dysregulated estrogen signaling, particularly the imbalance between estrogen receptor subtypes, plays a central role in driving these processes and may contribute to the persistence and progression of ectopic lesions. In parallel, also the involvement of stem or progenitor cells has been highly investigated because they may support lesion establishment, cellular plasticity, and long-term disease maintenance. These mechanisms overlap with pathways commonly involved in tumor initiation and progression. Recognizing endometriosis as a stem cell-driven and estrogen-dependent condition, the perspective, in both clinical management and therapeutic strategies fields, can change. Indeed, it is essential to emphasize that endometriosis is a benign condition and that the risk of developing an associated tumor is very low, approximately 1.5–2%. This review aims to discuss current evidence on the molecular aspects, focusing on estrogen signaling, stem cell-related mechanisms, and inflammatory and microenvironmental pathways that contribute to disease development. By highlighting these mechanisms, an integrated perspective on the pathophysiology of endometriosis is provided, also to outline potential implications for biomarker discovery and targeted therapeutic strategies.

## 1. Introduction

Endometriosis is a chronic, estrogen-dependent inflammatory disorder characterized by the presence of endometrial-like tissue outside the uterine cavity. It affects approximately 10% of women of reproductive age and is commonly associated with debilitating symptoms such as chronic pelvic pain, dysmenorrhea, dyspareunia, urinary discomfort, infertility, and fatigue [1]. Despite its high prevalence and clinical impact, the pathogenesis of endometriosis remains only partially understood. Several hypotheses have been proposed, including retrograde menstruation, coelomic metaplasia, and stem cell-related mechanisms, but none fully explain the origin and persistence of ectopic lesions [2,3].

A hallmark of endometriosis is the establishment of ectopic implants capable of surviving, proliferating, and invading surrounding tissues [4,5]. These lesions are composed of both glandular epithelial and stromal cells and are sustained by a complex microenvironment characterized by chronic inflammation, enhanced vascularization, and immune dysregulation [6]. Although the uterus is structurally present at birth, it undergoes profound morphological and cellular changes during development and puberty, suggesting that alterations in developmental pathways may contribute to the ectopic establishment of endometrial-like cells [7]. Clinically, endometriosis is frequently diagnosed during infertility investigations, although the causal relationship between the disease and impaired fertility remains debated. Concerning the best diagnostic strategy, laparoscopic visualization is still considered the gold standard, even if it is an underestimation of disease prevalence in asymptomatic women that should be considered [8].

Endometriosis is strongly influenced by estrogen signaling. An imbalance between estrogen receptor subtypes—characterized by increased expression of Estrogen Receptor β (*ERβ*) and suppression of Estrogen Receptor α (*ERα*)—results in an elevated *ERβ/ERα* ratio that promotes lesion survival and progression [9]. *ERβ* has been shown to regulate key cellular processes including apoptosis, mitochondrial function, oxidative stress responses, and inflammatory signaling pathways [10]. Through the modulation of factors such as *NRF1*, *SOD2*, *COX-2*, and Matrix Metalloproteinases, *ERβ* contributes to a permissive microenvironment that favors cellular proliferation, tissue invasion, and lesion persistence [11,12]. Although endometriosis is traditionally classified as a benign disease, accumulating evidence indicates that it shares several molecular and biological characteristics with malignant processes. These include enhanced proliferative capacity, resistance to apoptosis, increased angiogenesis, local invasion, and the ability to remodel the surrounding microenvironment through chronic inflammation and immune escape mechanisms [13]. In addition, prolonged exposure to oxidative stress and inflammatory mediators may promote genetic and epigenetic alterations that overlap with those observed in certain cancers [14,15]. Notably, Ovarian Endometriosis (OE) has been associated with an increased risk of developing specific malignancies, such as Clear Cell Ovarian Carcinoma (OCCC) and Endometrioid Ovarian Carcinomas (EOvCs) [16]. In this context, increasing attention has been directed toward the role of stem or progenitor cells and their potential contribution to lesion initiation, cellular plasticity, and long-term disease persistence [17,18]. These mechanisms, together with dysregulated estrogen signaling and microenvironmental alterations, suggest that endometriosis may display several biological features reminiscent of tumorigenesis.

This review discusses the molecular mechanisms linking endometriosis to cancer-like processes, with particular focus on the role of estrogen signaling, stem cell plasticity, and microenvironmental factors that contribute to disease development and progression. By integrating current knowledge on these converging pathways, we aim to provide a conceptual framework that may explain the persistence and the complexity of endometriosis and highlight potential implications for future therapeutic strategies.

### Search Strategy and Selection Criteria

To ensure a balanced and objective synthesis of the molecular and biological events observed during endometriosis and those occurring in malignancy, a comprehensive literature search was conducted across PubMed, Scopus, and Web of Science databases. The research focused primarily on peer-reviewed articles published between 2010 and 2024; fundamental studies on the pathogenesis of endometriosis were included regardless of their publication date, instead.

The selection of literature followed a clear hierarchy of evidence: (1) epidemiological risk: To assess the risk of malignant transformation (Endometriosis-Associated Ovarian Cancers, EAOCs), we prioritized prospective cohort studies and meta-analyses over case reports, to avoid overestimating the absolute risk, which remains approximately 1.5–2%; (2) molecular mechanisms: For “cancer-like hallmarks” such as invasiveness, neo-angiogenesis, and immune evasion, evidence was integrated from both human tissue studies (e.g., ERβ/ERα expression profiles) and preclinical experimental models (e.g., cell lines and animal models) to describe the signaling pathways involved; (3) genetic profiling: Studies identifying somatic mutations (e.g., *ARID1A*, *PIK3CA*, *KRAS*) were selected based on their focus on the distinction between benign lesions and those showing atypical features; (4) therapeutic strategies: The discussion on targeted therapies (e.g., PI3K/mTOR or PARP inhibitors) was limited to those supported by preclinical evidence or ongoing clinical trials, explicitly acknowledging the translational gap between oncological models and benign chronic disease.

## 2. Estrogen Signaling Reprogramming in Endometriosis

### 2.1. ERβ Overexpression

One of the most distinctive molecular features of endometriotic lesions is the marked overexpression of ERβ compared with eutopic endometrium. This alteration represents a key event in the reprogramming of estrogen signaling and plays a central role in the pathophysiology of the disease. Studies have consistently demonstrated that *ERβ* levels are significantly higher in ectopic endometrial tissues, whereas *ERα* expression is relatively reduced, resulting in a dysregulated *ERβ/ERα* ratio [19,20,21,22]. *ERβ* overexpression contributes to lesion survival through multiple mechanisms. At the transcriptional level, *ERβ* modulates the expression of genes involved in apoptosis resistance, inflammation, and cellular proliferation. Specifically, *ERβ* has been shown to inhibit apoptotic pathways by repressing pro-apoptotic signaling and promoting cell survival, thereby facilitating the persistence of ectopic endometrial cells [22]. In addition, *ERβ* enhances inflammatory responses by upregulating mediators such as *CycloOXygenase-2* (*COX-2*), which in turn stimulates ProstaGlandin E2 (PGE2) production and further increases local estrogen biosynthesis [23]. Beyond its role in transcriptional regulation, *ERβ* also participates in non-genomic signaling pathways that influence cellular adaptation and microenvironmental interactions. Emerging evidence suggests that *ERβ* acts in multiple intracellular signaling cascades, including pathways involved in immune modulation and cellular stress responses, thereby contributing to the establishment of a permissive niche for lesion growth and invasion [20,24]. Recent studies have further highlighted the role of *ERβ* as a key regulator of the inflammatory and proliferative phenotype of endometriotic cells. *ERβ* has been implicated in the modulation of immune cell recruitment and cytokine production, as well as in the regulation of genes associated with cellular plasticity and survival, reinforcing its central role in disease progression [25]. Overall, ERβ overexpression represents a central driver of endometriosis progression, promoting key biological processes such as proliferation, resistance to apoptosis, and inflammation. Notably, these mechanisms overlap with pathways commonly observed in tumorigenesis, reinforcing the concept that endometriosis shares several cancer-like molecular features.

### 2.2. ERα Suppression

In parallel with *ERβ* overexpression, endometriotic lesions are characterized by a significant suppression of *ERα*, which plays a crucial role in the physiological regulation of endometrial function. The downregulation of *ERα* contributes to the disruption of normal estrogen signaling and further amplifies the imbalance between *ERβ* and *ERα*, a key feature of endometriosis pathophysiology [19,20,21,22]. *ERα* is the predominant estrogen receptor in the eutopic endometrium and is primarily responsible for mediating the proliferative and differentiative effects of estrogen under physiological conditions. Its reduced expression in ectopic lesions leads to an altered transcriptional response to estrogen, favoring *ERβ*-driven signaling pathways that promote cell survival, inflammation, and tissue invasion [20,21]. This shift in receptor dominance results in a loss of regulatory balance and contributes to the persistence of endometriotic tissue. The mechanisms underlying *ERα* suppression in endometriosis are complex and involve both genetic and epigenetic factors. In particular, hypermethylation of the *ESR1* gene promoter has been identified as a key mechanism responsible for decreased *ERα* expression in ectopic endometrial cells [22,26]. Epigenetic silencing of *ERα* not only alters estrogen responsiveness but also contributes to the establishment of a stable, disease-specific transcriptional profile that supports lesion maintenance and progression. Furthermore, *ERα* downregulation has been associated with progesterone resistance, a well-recognized feature of endometriosis [27]. The loss of *ERα*-mediated signaling impairs progesterone receptor expression and function, thereby disrupting hormonal responsiveness and further promoting a pro-inflammatory and proliferative environment [20,28]. This hormonal imbalance contributes to the chronicity of the disease and reduces the effectiveness of conventional hormonal therapies. Recent evidence suggests that *ERα* suppression may also enhance cellular plasticity and facilitate the acquisition of a more aggressive phenotype. The loss of *ERα*-dependent regulatory pathways, combined with *ERβ* predominance, promotes signaling networks involved in inflammation, extracellular matrix remodeling, and resistance to apoptosis, thereby reinforcing biological processes that resemble those observed in tumorigenesis [20,24,28]. Taken together, the suppression of *ERα* represents a critical component of estrogen signaling reprogramming in endometriosis. In combination with *ERβ* overexpression, it contributes to a dysregulated hormonal environment that favors lesion persistence, immune evasion, and invasive behavior, further supporting the concept of endometriosis as a disease with cancer-like molecular features.

### 2.3. Mitochondrial Signaling

In addition to its transcriptional activity, *ERβ* plays a critical role in the regulation of mitochondrial function and cellular metabolism in endometriotic cells. Mitochondria are central regulators of cellular energy production, redox balance, and apoptotic signaling, and their dysfunction has emerged as a key feature also in the pathophysiology of endometriosis [20,29]. *ERβ* has been shown to directly influence mitochondrial gene expression and bioenergetic processes. Through these mechanisms, *ERβ* modulates mitochondrial biogenesis, respiratory chain activity, and the production of Reactive Oxygen Species (ROS), thereby contributing to metabolic reprogramming in ectopic endometrial cells [20,30]. This altered mitochondrial function enables cells to adapt to the hostile microenvironment of ectopic lesions, characterized by hypoxia, inflammation, and oxidative stress. Recent evidence indicates that mitochondrial dysfunction in endometriosis is closely associated with increased ROS production and impaired antioxidant defenses. Mitochondria-derived ROS act not only as damaging agents but also as signaling molecules that activate pathways involved in cell proliferation, survival, and inflammation [31,32]. Indeed, oxidative stress-induced activation of signaling cascades such as MAPK and mTOR pathways has been shown to promote the growth and persistence of endometriotic lesions [32]. Furthermore, mitochondrial alterations contribute to resistance to apoptosis, a hallmark of endometriosis. Dysregulated mitochondrial signaling can impair the intrinsic apoptotic pathway, allowing ectopic endometrial cells to evade programmed cell death and survive in ectopic sites [20,33]. This anti-apoptotic phenotype is further reinforced by *ERβ*-mediated signaling, which interacts with mitochondrial pathways to sustain cell viability under stress conditions. Emerging studies have also highlighted the interplay between mitochondrial dysfunction, iron metabolism, and ferroptosis in endometriosis. Iron overload in the peritoneal environment promotes mitochondrial ROS generation and lipid peroxidation, while endometriotic cells develop adaptive mechanisms to resist ferroptotic cell death, further contributing to lesion persistence [31]. Overall, mitochondrial signaling represents a crucial component of estrogen-driven cellular reprogramming in endometriosis. By regulating energy metabolism, oxidative stress, and apoptotic resistance, mitochondrial dysfunction contributes to the acquisition of a phenotype characterized by enhanced survival, adaptability, and proliferation. Notably, these features closely resemble metabolic, and survival strategies observed in tumor cells, supporting the concept of endometriosis as a disease with cancer-like biological behavior.

### 2.4. Oxidative Stress

Oxidative stress represents a key pathogenic mechanism in endometriosis, and it is closely interconnected with estrogen signaling and mitochondrial dysfunction. It is defined as an imbalance between the production of ROS and the capacity of antioxidant defense systems, leading to cellular damage and altered signaling pathways [28,34]. In endometriosis, elevated levels of ROS have been consistently detected in the peritoneal fluid and ectopic lesions, reflecting a pro-oxidant microenvironment that contributes to disease progression [34,35]. Several sources contribute to oxidative stress in endometriosis, including retrograde menstruation, chronic inflammation, and mitochondrial dysfunction. The accumulation of erythrocytes and iron in the peritoneal cavity promotes the generation of free radicals through Fenton reactions, further amplifying oxidative damage [36,37]. In addition, *ERβ*-driven signaling has been shown to modulate oxidative stress responses by regulating genes involved in redox balance, thereby linking hormonal dysregulation to ROS production [28,35]. Beyond its damaging effects, oxidative stress plays a crucial role as a signaling mediator in endometriotic cells. ROS can activate multiple intracellular pathways, including MAPK, NF-κB, and PI3K/AKT signaling cascades, which are involved in cell proliferation, survival, inflammation, and angiogenesis [38]. These pathways contribute to the establishment of a pro-survival phenotype and enhance the invasive potential of ectopic endometrial tissue. Oxidative stress is also closely associated with epigenetic alterations in endometriosis. ROS-induced DNA damage and aberrant methylation patterns can lead to the dysregulation of genes involved in hormonal signaling, inflammation, and cellular differentiation [39,40]. In particular, oxidative stress has been implicated in the epigenetic silencing of key regulatory genes, further stabilizing the pathological phenotype of endometriotic lesions. Recent studies have highlighted the interplay between oxidative stress, lipid peroxidation, and ferroptosis in endometriosis. Although increased ROS levels would be expected to induce ferroptosis, endometriotic cells appear to develop adaptive mechanisms that confer resistance to this cell death program, allowing them to survive in a highly oxidative environment [41,42]. This resistance further contributes to lesion persistence and mirrors similar survival strategies observed in cancer cells. Furthermore, oxidative stress is tightly linked to immune dysregulation and chronic inflammation, creating a self-sustaining pathogenic loop. ROS-mediated activation of inflammatory pathways promotes cytokine release and immune cell recruitment, thereby amplifying the inflammatory microenvironment that characterizes endometriosis [43]. This interaction between oxidative stress and inflammation further enhances tissue remodeling, angiogenesis, and lesion progression. Overall, oxidative stress represents a central hub in the pathophysiology of endometriosis, integrating hormonal, metabolic, and inflammatory signals. By promoting DNA damage, epigenetic reprogramming, and activation of pro-survival pathways, oxidative stress contributes to the acquisition of biological features such as enhanced proliferation, resistance to apoptosis, and tissue invasion. These processes closely resemble mechanisms involved in tumorigenesis, further supporting the concept of endometriosis as a disease with cancer-like characteristics [28,44].

### 2.5. Inflammation

Chronic inflammation is a defining feature of endometriosis and plays a central role in the establishment, progression, and persistence of ectopic lesions. The peritoneal environment of affected women is characterized by increased levels of pro-inflammatory cytokines, chemokines, and growth factors, which collectively create a permissive niche for lesion survival and expansion [28,44]. This inflammatory milieu is sustained by complex interactions between endometrial cells, immune cells, and the surrounding microenvironment. A key component of this process is the dysregulation of immune surveillance. Endometriotic lesions are associated with altered immune cell function, including impaired cytotoxic activity of natural killer (NK) cells, increased numbers of activated macrophages, and the recruitment of regulatory T cells (Tregs), all of which contribute to immune tolerance and allow ectopic cells to evade clearance [44,45]. Specifically, macrophages play a pivotal role by secreting pro-inflammatory cytokines such as IL-6, TNF-α, and IL-1β, as well as angiogenic factors that promote lesion vascularization and growth. Estrogen signaling, especially through *ERβ*, is closely intertwined with inflammatory pathways. *ERβ* activation enhances the expression of inflammatory mediators, including COX-2 and PGE2, which, in turn, stimulate local estrogen production and create a positive feedback loop that sustains both inflammation and hormonal dysregulation [46]. This bidirectional interaction between estrogen signaling and inflammation represents a key mechanism, driving disease chronicity. Oxidative stress further amplifies the inflammatory response by activating redox-sensitive signaling pathways such as NF-κB and MAPK, leading to increased cytokine production and immune cell recruitment [38,47]. The interplay between ROS and inflammatory signaling contributes to the establishment of a self-perpetuating pathogenic cycle in which oxidative stress and inflammation reinforce each other, promoting tissue damage and lesion progression. In addition to sustaining inflammation, the endometriotic microenvironment actively supports angiogenesis and extracellular matrix remodeling. Pro-inflammatory mediators stimulate the expression of Vascular Endothelial Growth Factor (VEGF) and Matrix Metalloproteinases (MMPs), facilitating neovascularization and tissue invasion [24,28]. These processes are essential for the maintenance and expansion of ectopic lesions and closely resemble mechanisms observed in tumor progression. Recent evidence highlights that chronic inflammation in endometriosis is not merely a bystander phenomenon but a driver of cellular plasticity and disease evolution. Inflammatory signaling pathways can induce phenotypic changes in endometrial cells, promoting survival, migration, and adaptation to ectopic environments [48,49]. Moreover, the inflammatory microenvironment contributes to epigenetic modifications and immune escape mechanisms, further stabilizing the pathological state. Overall, inflammation represents a central hub in the pathophysiology of endometriosis, integrating hormonal, metabolic, and immune signals. The persistent activation of inflammatory pathways promotes key biological processes—including proliferation, angiogenesis, immune evasion, and tissue invasion—that closely resemble cancer-associated hallmarks. These observations reinforce the concept of endometriosis as a chronic inflammatory disease with tumor-like biological behavior.

## 3. Endometriosis, Adenogenetic Factors and Estrogen-Driven Uterine Remodeling

Functional convergence of different biological events is observed in endometriosis.

Specifically, hormonal imbalance, inflammatory signaling, and stress-adaptive pathways act cooperatively to promote lesion survival under hostile ectopic conditions, generating selective pressures that favor cellular plasticity and persistence [4,50,51,52,53]. In this context, the adaptive intent is clear, rather than an oncogenic one, providing a biological explanation about the occurrence of malignant transformation only in some cases.

Uterine adenogenesis represents a fundamental developmental process regulated by a complex network of hormonal, genetic, and paracrine factors that ensure the correct formation and functionality of endometrial glands. Several genes—including *FOXA2*, *WNT4*, *WNT5A*, *WNT7A*, and *E-CaDHerin* (*CDH1*)—play crucial roles in glandular morphogenesis and epithelial–stromal communication. Experimental models have shown that the conditional deletion of *FOXA2* or *WNT* family members results in a marked reduction or complete absence of uterine glands, confirming their indispensable role in maintaining uterine homeostasis and fertility [54,55]. These adenogenetic pathways are tightly modulated by ovarian hormones, particularly estradiol-17β and prolactin, which orchestrate epithelial proliferation, extracellular matrix remodeling, and the differentiation of glandular epithelium during postnatal uterine development [56].

Emerging evidence indicates that dysregulation of adenogenetic signaling contributes to the establishment and persistence of endometriotic lesions. Immunohistochemical studies have demonstrated that FGF7, FGF10, and HGF are significantly downregulated in the epithelium and stroma of endometriosis tissues, as compared to eutopic endometrium; FGF23 and IFN-τ, instead, are markedly overexpressed in ectopic stromal compartments [57,58]. These alterations suggest a shift in epithelial–mesenchymal signaling, impairing normal glandular homeostasis and favoring ectopic gland survival under estrogenic stimulation. Aberrant activation of these pathways may also enhance cellular motility, invasiveness, and local angiogenesis, thus promoting a microenvironment that mimics the tumorigenic behavior observed in EAOCs. Collectively, these findings highlight that adenogenetic factors—traditionally linked to uterine development—may also mediate estrogen-driven remodeling and neoadenogenesis in endometriosis and potentially lead to pre-malignant tissues.

In Table 1 a list of adenogenetic factors involved in both endometriosis and ovarian cancers has been reported. Unfortunately, these factors have a potential role in oncogenic transformation, supporting the hypothesis that, only in a small cohort of cases, aberrant adenogenetic signaling, together with the convergence of other conditions, such as estrogen signaling dysregulation, may facilitate tumor progression.

Collectively, the activation of cancer-related signaling pathways in endometriosis reflects an adaptive response to ectopic stress rather than a linear progression toward malignancy. These mechanisms enhance cellular fitness, immune evasion, and tissue remodeling, creating a biological substrate that only rarely, and under additional genomic or microenvironmental constraints, may permit malignant evolution. Therefore, the adenogenetic factors represent a dual function in both physiological gland development and pathological proliferation which, only in multifactorial convergence of conditions, may play a significant positive role in the tumorigenesis.

## 4. Cancer-like Hallmarks in Endometriosis

Despite the benign nature of the disorder, endometriosis shares multiple biological and molecular characteristics with malignant tumors. Among the most notable parallels, there are tissue invasiveness, neo-angiogenesis, resistance to apoptosis, and a pro-inflammatory microenvironment, all key hallmarks of cancer. Endometriotic lesions can infiltrate surrounding tissues, establishing their own blood supply and persisting, despite physiological mechanisms that would typically induce cell death.

At the molecular level, endometriosis shares key genetic and epigenetic alterations with cancer. Changes in DNA methylation patterns, histone modifications, and non-coding RNA expression contribute to the dysregulation of gene expression, inflammation, and immune escape. Genome-Wide Association Studies (GWAS) have identified several susceptibility loci for endometriosis, with most variants located in non-coding regions, suggesting a role in transcriptional regulation rather than protein structure [69,70,71]. For example, the 9p21 risk locus was shown to alter gene expression through a cascade, involving transcription factor binding and chromatin interactions [72].

Somatic mutations commonly associated with cancer, such as those in ARID1A, PIK3CA, and PTEN, have also been found in ovarian endometriotic lesions, particularly those linked to OCCC and EOvC (see Table 2) [73,74].

However, depending on the tumor subtype (OCCC vs. EOvC), there is a wide mutation frequency of the above-mentioned genes. For example, *ARID1A* is mutated in approximately 50% of patients with OCCC, while *PIK3CA* varies between 30 and 40% for the same subtype [76,77,78,79]. EOvC subtype reports *CTNNB1* as one of the most frequently altered targets in endometrioid tumors, with percentages of approximately 40% of patients [79,80]. Mutation frequencies for *PTEN* and *KRAS*, instead, are variable, showing *PTEN* loss/mutations in EOvC in ~20–45% of cases, while *KRAS* is frequently mutated in both endometriosis and endometrioid tumors, with percentages that can reach 60% [80,81,82,83]. However, it should be noted that direct causality depends on the concurrence of genomic and microenvironmental factors, as demonstrated in experimental models [82,83,84]. It is fundamental to point out that if these mutations contribute to an increased risk of malignant transformation, the absolute risk of developing forms of cancer from endometriotic lesions remains low.

As already mentioned, besides genetic alterations, other mechanisms—such as changes in gene expression through epigenetic regulation (i.e., DNA methylation)—may have a crucial role in the progression of endometriosis, to its malignant transformation [85]. For example, chronic inflammation and cyclic regeneration may induce inactivation or epigenetic silencing of the *ARID1A* gene in the epithelial component of endometriosis [86,87]. Also, activation of the PI3K/AKT pathway was described in endometriosis because of reduced decidualization both in endometriotic lesions and in eutopic endometrium of patients with endometriosis [88,89]. Another epigenetic regulation concerns *GATA2* and *GATA6* genes: different DNA methylation of these two genes has been observed in endometriosis. The abundant and unmethylated *GATA2* was found in stromal cells of eutopic endometrium, as well as the methylated and inactive form in stromal cells of endometriosis. *GATA6*, instead, was found to be methylated and inactive in eutopic endometrium stromal cells and active and unmethylated in endometriosis stroma [90]. The overexpression of *GATA6* in endometriosis causes, in turn, different levels of hormone receptors expression, with reduction in *ERα* and PR, stimulation of *Erβ*, which contribute to the altered *ERβ/ERα* ratio [19].

**Figure 1:**
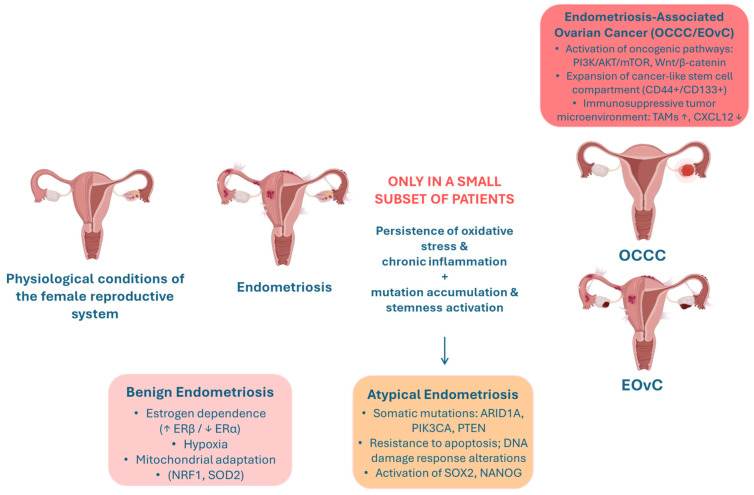
Key molecular events which, in only a few cases, may explain the transition of benign endometriosis to EAOCs, such as OCCC and EOvC. Progression is driven by chronic inflammation and oxidative stress, accumulation of driver mutations (ARID1A, PIK3CA, PTEN), and activation of stemness and oncogenic pathways (PI3K/AKT, Wnt). TAMs = Tumor-Associated Macrophages.

These findings support the hypothesis that a small subset of cases, especially those involving the ovary, may represent the precursor conditions to malignant transformation (Figure 1).

However, detecting somatic mutations in endometriotic tissue poses technical challenges due to the high proportion of stromal and immune cells in lesions. To address this issue, laser microdissection has been used to isolate epithelial cells for next-generation sequencing analysis, allowing more precise characterization of genetic alterations [91].

Estrogens, especially estradiol, contribute to the tumor-like behavior of endometriosis by promoting inflammation, cell survival, and tissue remodeling through the overexpression of *ERβ* [10].

In addition to molecular and hormonal similarities, deep endometriosis (DE), a severe phenotype, has been increasingly linked to a higher risk of malignancy. Advances in imaging have allowed for non-invasive diagnosis and the monitoring of DE. Despite its aggressive clinical behavior and cancer-like infiltration of pelvic organs, approximately 50% of DE does not proceed through malignant transformation, and hormonal therapies (in particular, combined oral contraceptives and progestins), have been shown to significantly reduce progression. These treatments can decrease lesion size and improve quality of life, even without measurable size reduction [92]. This clinical behavior, coupled with molecular parallels, underscores the importance of further investigation into the oncogenic potential of endometriosis and the development of targeted prevention strategies. Nevertheless, the heterogeneity of associations between the macrophenotypic subtypes of endometriosis (superficial peritoneal endometriosis, ovarian endometriomas and deep infiltrating endometriosis) and histotypes of ovarian carcinoma are very complex [93,94]. The comparison of endometriosis subtypes reveals distinct molecular landscapes and varying degrees of malignant risk. Indeed, even if with a very low rate, DE is not the only phenotype that appears to be associated with an increased risk of developing a malignant lesion. Also, ovarian endometriomas represent the primary precursor to EAOC. Indeed, according to Prat J., EOvCs and OCCCs would be associated with a previous endometriotic condition [95,96]. This transition is driven by the unique microenvironment of the ovarian cyst, where high concentrations of free iron from recurrent hemorrhage induce oxidative DNA damage and specific mutations in *ARID1A* and *PIK3CA* [84,97,98,99].

Specifically, the transition process from benign endometriosis to carcinoma would be interspersed with atypical endometriosis, in which the lesion acts as a precursor to endometrioid and OCCCs [100,101].

Barnard ME et al. highlights how women affected by endometriosis have a 4.2 times higher risk of ovarian cancer than those not affected; patients with ovarian endometriomas and/or deep infiltrating endometriosis reported a 9.7 times higher risk than those not affected by endometriosis, instead. Even more interestingly, it has been observed that there is a higher frequency of associations between endometriosis subtypes and ovarian cancer histotypes for type I ovarian cancer (endometrioid, clear cell, mucinous, and low-grade serous) than for type II ovarian cancer (high-grade serous) [102].

Additionally, recent studies have increasingly focused on the role of Endometriosis Stem Cells (ESCs) in the pathogenesis of EAOC, suggesting that their self-renewal capacity, resistance to apoptosis, and susceptibility to genetic and epigenetic alterations may predispose them to malignant transformation [103]. Although Wilczyński JR et al. do not report specific gene mutations, extensive literature supports the recurrent involvement of several oncogenic and tumor suppressor genes in EAOC. Notably, mutations in *ARID1A*, a chromatin remodeling gene, are found in approximately 45–54% of OCCC and EOvC, often in conjunction with activating mutations in *PIK3CA* or loss of *PTEN*, which together dysregulate the *PI3K/AKT/mTOR* signaling pathway [84]. *KRAS* mutations, commonly associated with low-grade serous and EOvC, as well as alterations in *TP53*, *CTNNB1* (Wnt/β-catenin pathway), and *HNF1B*, further highlight the heterogeneous molecular landscape of EAOC. Yong Song et al. investigated the expression of stemness-related genes, specifically the pluripotency-associated transcription factors *OCT4*, *SOX2*, and *NANOG*, in women with endometriosis [104]. These genes are key regulators of self-renewal and pluripotency in embryonic stem cells and primordial germ cells. The study found that their expression levels were significantly elevated in women with endometriosis as compared to healthy controls. Notably, both mRNA and protein levels of *SOX2* were markedly higher in the eutopic endometrium of affected individuals, further supporting the hypothesis that endometriosis may originate from cells with stem-like properties. In his thesis on endometriosis, Di Claudio highlighted the potential to identify a subgroup of patients at higher risk for malignant transformation, characterized by the significant upregulation of genes involved in cellular reprogramming (such as *SOX2* and *NANOG*), cancer metabolism (*TP53*, *KRAS*), and the Epithelial–Mesenchymal Transition (*TGF-α*, *SNAI1*) [105]. Moreover, in 3D spheroid cultures derived from endometriotic tissue, there was an increased co-expression of Cancer Stem Cell (CSC) surface markers CD44 and CD133, particularly in the high-risk group, which was also associated with enhanced invasive capacity. These findings support a possible link between endometriosis and its malignant potential, providing insights into the mechanisms underlying endometriosis-associated pathogenesis. These genetic changes, often detectable in endometriotic lesions adjacent to tumors, support the theory of a stepwise progression from benign endometriosis to malignancy, with ESCs as a plausible cellular origin, in line with evidence suggesting that endometriotic cells may derive from developmental remnants present since fetal life [106]. It is fundamental to note that, although the stem cell hypothesis is well established, much of the evidence in humans remains only correlational, while causal evidence is derived mainly from functional studies performed on animal models [107].

Thus, while endometriosis does not typically progress to cancer, the shared molecular architecture with malignancies raises important questions about its oncogenic potential and long-term risks. These parallels support the use of cancer-derived molecular tools (e.g., next-generation sequencing, laser microdissection) for studying endometriosis, also suggesting that revisited targeted therapies may offer new treatment strategies for this chronic and often refractory disease, with limited adverse effects.

Lastly, recent advancements in non-invasive diagnostics have shifted focus toward the systemic immune response to endometriotic lesions. Beyond traditional protein biomarkers, autoantibody screening represents a promising frontier, reflecting the immune system’s recognition of altered cellular proteins within the ectopic microenvironment. A pivotal large-scale study by Laudański et al. conducted a comprehensive autoantibody screening of plasma and peritoneal fluid in patients with endometriosis, using high-throughput protein microarrays to screen over 21,000 human proteins [108]. This proteome-wide approach identified a distinct autoantibiome featuring 59 differentially prevalent autoantibodies in patients compared to controls. Notably, the study demonstrated that specific autoantibody panels could distinguish patients with high diagnostic accuracy, highlighting the potential of the humoral immune response for early detection. This approach parallels early-detection strategies in oncology, as precursors to clinical manifestation, potentially offering a powerful tool to overcome the diagnostic delay inherent in surgical laparoscopy.

Collectively, estrogen signaling, immune dysfunction, stem cell-related plasticity, adenogenic processes, and microenvironmental pressures should be interpreted as interacting components of a unified adaptive system, rather than parallel oncogenic pathways. Indeed, estrogen dominance amplifies inflammatory signaling and promotes survival pathways, while compromised immune surveillance reduces the effectiveness of lesion clearance. In this context, chronic inflammation and tissue damage favor epigenetic and stemness-associated programs that increase cellular plasticity, regeneration, and resistance to apoptosis. At the same time, repeated cycles of tissue remodeling and adenogenesis within ectopic sites expose cells to hypoxia, oxidative stress, and mechanical constraints conditions, further reinforcing stress adaptation signaling. These pressures do not inherently confer malignant intent but support lesion persistence and heterogeneity. When this adaptive landscape is combined with permissive genetic or epigenetic alterations, the risk of malignant transformation increases.

Thus, although endometriosis recapitulates several hallmarks of cancer, it remains a benign, non-clonal and non-metastatic condition and the overlap in molecular pathways suggests shared biological and molecular circuitries. In this integrated context, the nature of endometriosis-associated neoplasms is conditional and not deterministic.

## 5. Endometriosis-Associated Malignancies and the Role of the Microenvironment

Many are the molecular alterations associated with endometriosis. Here, they are represented as components of a dynamic process shaped by chronic inflammation, hormonal imbalance, hypoxia, and immune pressure, rather than static abnormalities, genetic, epigenetic, and/or signaling alterations. This perspective allows us to emphasize temporal disease evolution and microenvironment-driven selection, in which persistence, in rare cases, may lead to malignant progression.

Indeed, endometriosis is a benign disease; nonetheless, a small subgroup of lesions, such as ovarian endometriomas, can follow biological pathways that lead to EAOC. Thus, integrated aspects, such as retrograde menstruation, local hormonal imbalances, immune dysfunction, stem cell-like cell phenotypes, and microenvironment remodeling, may explain the occurrence of this transformation and progression towards greater lesion aggressiveness [108]. Specifically, retrograde menstruation causes the presence of endometrial cells in the pelvic cavity, and alterations in the innate and acquired components of the immune system create a favorable microenvironment for the persistence of the lesion [6,8]. In addition, the dysregulation of estrogen signaling (i.e., dominant activity of *ERβ*) promotes the infiltration of inflammatory cells into the ectopic tissue and the evolution of the lesion [109]. At the same time, a subpopulation of epithelial/stromal cells with stem cell characteristics, such as self-renewal capacity, activation of Wnt/β-catenin pathways and autophagy, contributes to the persistence of the lesion, conferring resistance to conventional hormonal therapies.

Repeated events of inflammation, hypoxia, and matrix remodeling create selective pressures whereby cells harboring somatic driver alterations (e.g., *ARID1A*, *PIK3CA*, *PTEN*, *CTNNB1*) undergo clonal expansion, fixing those genetic traits in EAOC lesions [84]. It is important to note that the acquisition of the mutation alone is generally insufficient for malignant conversion; indeed, malignancy appears to require cooperation between genetic factors and a permissive hormonal/immune microenvironment. A recent study investigated the molecular differences between endometriosis and *EAOC*, focusing on autophagy-related genes. Analysis of gene expression data identified *CXCL12* as a key differentially expressed gene. *CXCL12* expression was consistently lower in cancerous tissues, including *EAOC*, and was associated with worse prognosis, tumor stage, immune subtype, and molecular classification across multiple cancers. Immunohistochemical analysis revealed that CXCL12, IL17, STAT3, FOXP3, and the Th17/Treg ratio were all reduced in EAOC, as compared to endometriosis and normal endometrial tissues. These findings suggest that CXCL12 downregulation and immune imbalance may contribute to the progression from endometriosis to EAOC and support its potential role as a prognostic marker in cancer [110]. Recent research has also focused on microRNAs (miRNAs) as regulators of gene expression involved in malignant transformation. A study profiling miRNA in benign OE and EAOC identified a panel of miRNAs—such as *miR-200a-3p*, *miR-141-3p*, *miR-183-5p*, and *miR-10a-5p*—that are significantly upregulated in malignant and at-risk tissues. These miRNAs demonstrated high diagnostic accuracy, highlighting their potential as early biomarkers for detecting malignant progression in OE [111]. Recent advances have shed light on the molecular mechanisms underlying the development of Endometriosis-Related Ovarian Neoplasms (ERONs) from benign endometrioma, with particular focus on their progression into either EOvC or OCCC subtypes. A novel study employed in vitro and in vivo models reported the use of immortalized epithelial cells derived from endometrioma tissue, manipulated through the overexpression or knockout of key genetic drivers such as *ARID1A*, *KRAS*, *AKT*, and *MYC*. The combination of *ARID1A* loss with either *KRAS* or *AKT* activation and c-Myc overexpression proved to be sufficient to induce malignant transformation in immunocompromised mice. Notably, the resulting tumor histology varied depending on the host immune environment: OCCC developed in SCID mice, while EOvC appeared in nude mice. These findings suggest a critical role for the tumor immune microenvironment, particularly B-cell signaling, in shaping ERON histotypes. This model provides a valuable tool to further dissect the molecular pathways of ERON carcinogenesis and offers potential for identifying novel therapeutic targets [112]. A rare but illustrative clinical case further highlights the malignant potential of endometriosis [113]. A 46-year-old woman with a long-standing history of surgically confirmed endometriosis and persistent abdominal pain was found to have a cystic mass in the left mesogastrium. Surgical excision followed by histological examination confirmed a low-grade EOvC arising from extragenital endometriosis. The patient underwent radical surgery and adjuvant chemotherapy, achieving complete remission. This case underscores the diagnostic challenges and the need for oncologic vigilance, particularly when endometriosis is present in atypical locations or with suspicious clinical features.

**Figure 2:**
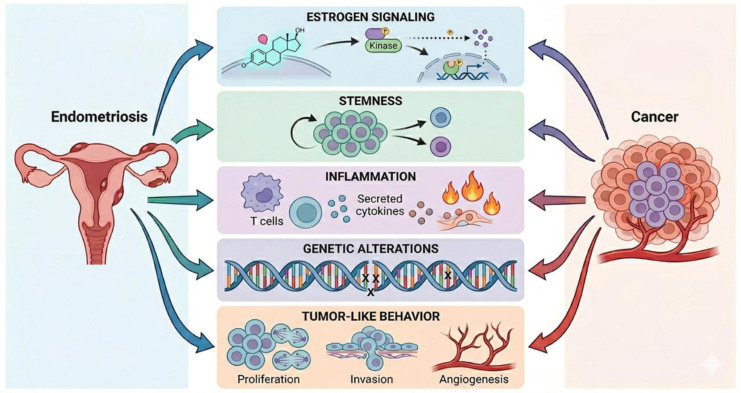
From Endometriosis to Neoplasia: Converging Molecular Mechanisms. Schematic representation of the key shared pathogenetic pathways between endometriosis and neoplastic diseases. The illustration highlights how both conditions are driven by overlapping cellular and molecular events. Specifically, this convergence encompasses the five primary domains, that are hormonal signaling, stemness, inflammation, genetic alterations and tumor-like behavior.

Taken together, the molecular alterations described in this section should be viewed as part of a time-dependent adaptive trajectory, in which chronic inflammation, hormonal signaling, and other microenvironmental stress and conditions progressively shape lesion biology (Figure 2).

While these processes may converge on pathways commonly associated with cancer, their primary role in endometriosis is to sustain survival and persistence. Only when additional genetic, epigenetic, and microenvironmental constraints accumulate, the adaptive landscape potentially shifts toward malignant transformation, underscoring the conditional nature of endometriosis-associated malignancies. Thus, the molecular alterations involved in endometriosis should not be interpreted as linear factors of malignant transformation, but rather as context-dependent traits under the microenvironmental selection over time. Although many endometriotic lesions share signaling, genetic, or epigenetic changes with cancer, it should be emphasized that most cases remain biologically constrained by intact differentiation programs, immune-mediated control, and limited clonal expansion. Indeed, oncogenic progression appears to occur only in a minority of lesions in which multiple factors converge, including prolonged inflammatory pressure, prolonged exposure to estrogen, compromised immune surveillance, and the accumulation of specific genomic or epigenomic alterations. These “multilayered” requirements explain why endometriosis is a predominantly benign disease, despite it sharing molecular characteristics with cancer. Therefore, endometriosis-associated neoplasms are rare outcomes of prolonged adaptive stress rather than inevitable disease trajectories.

## 6. Therapeutic Implications and Future Directions

The management of endometriosis has traditionally relied on a symptom-oriented approach, primarily focusing on hormonal suppression to induce lesion atrophy and surgical excision of ectopic tissue. However, the high recurrence rates and the contraceptive nature of current treatments underline a significant unmet need for therapies based on biological drivers of the disease. The cancer-like hallmarks—such as local invasiveness, epigenetic reprogramming, and immune evasion—have opened new avenues for pharmacological intervention, suggesting that some of the pathways successfully targeted in oncology might be repurposed also for endometriosis. Nevertheless, unlike malignant tumors, endometriosis is a chronic, non-lethal condition that predominantly affects women of reproductive age. Consequently, any proposed targeted therapy must balance high efficacy in lesion reduction with a stringent safety profile, particularly regarding ovarian reserve, oocyte quality, and potential teratogenicity.

In this section, we provide a hierarchical overview of therapeutic strategies, moving from established standard treatments of care to emerging molecular targets, distinguishing treatments supported by robust clinical evidence and those currently in the preclinical or speculative stages (Table 3).

Among the already mentioned hallmarks shared with malignant diseases, endometriosis shows resistance to standard treatments. Consequently, while surgery remains a mainstay in treating both conditions, the presence or absence of fatal outcomes affects the allocation of research funding and represents a significant economic and social burden, which calls for a re-evaluation of its perceived severity [119,120].

The similarities with malignant lesions open the possibility to bridge the translation gap with the emerging therapeutic strategies based on the biological mechanisms of endometriosis.

Concerning current and standard treatments of care for endometriosis, the management remains focused on hormonal suppression and surgical excision. These treatments address the estrogen-dependent nature of the disease without targeting its cancer-like invasive or genetic components. As already discussed, endometriotic lesions exhibit a predominance of ERβ over ERα, this contributes to aberrant COX-2 expression, inflammatory prostanoid production and extracellular matrix remodeling.

In addition, preclinical evidence is currently being gathered on repurposed oncological strategies. Indeed, several pathways shared with malignancy have been targeted using repurposed cancer drugs in animal models or in vitro studies. Thus, targeted therapies under investigation include VEGF inhibitors (e.g., bevacizumab), PARP inhibitors (e.g., olaparib, niraparib), MEK inhibitors, and PI3K/mTOR/Akt inhibitors, which not only hold potential in ovarian cancer but also exhibit beneficial effects in deep endometriosis models [121].

Therapies such as PI3K/mTOR/Akt and MEK inhibitors interfere with signaling cascades downstream of estrogen-driven molecular alterations, showing their applicability and relevance also in inflammatory and hormonal contexts rather than in cancer alone. Nonetheless, their clinical implementation is currently limited by potential systemic toxicity and adverse effects on ovarian reserve. Indeed, although agents such as Temsirolimus and Everolimus have been tested and, in selected settings, approved for malignant gynecologic tumors (e.g., endometrial cancer), PI3K or mTOR inhibitors unfortunately showed systemic metabolic effects, immunosuppression and interfere with ovarian function in animal models; thus, their toxicity profiles make their translation into a benign and fertility-relevant disease ethically and clinically problematic. To date, no PI3K/AKT/mTOR inhibitors have already undergone clinical trials for endometriosis, and their role is currently limited to mechanistic modeling and risk stratification rather than treatment [59,122].

As previously stated, endometriosis is characterized by a microenvironment enriched in cytokines (e.g., IL-6, TNF-α, G-CSF), ROS, and hypoxia-induced factors, supporting lesion survival and establishing a context tumor microenvironment (TME)-like, (i.e., macrophage-mediated angiogenesis and matrix remodeling) [123]. Similarly, the invasive capacity of endometriosis is supported by upregulated MMPs (MMP-2, MMP-7, MMP-9), hypoxic signaling, and macrophage-dependent pro-angiogenic factors. VEGF inhibitors (i.e., Bevacizumab), as anti-angiogenic agents, on one hand demonstrated efficacy in preclinical models and systemic toxicity, thromboembolic risk, and adverse reproductive effects; on the other hand, these side implications preclude long-term or routine use and emphasize the need for localized or transient modulation rather than a chronic systemic inhibition [28,124].

Therefore, some therapeutic strategies directly act on mechanisms recognized as central for the persistence of lesion. For instance, among the recognized targets, it is possible to include HIF-1α, NF-κB, β-catenin, or Tumor-Associated Macrophages (TAMs), while examples of related therapeutic approaches capable of addressing the inflammatory and microenvironmental biology of endometriosis are CSF-1R blockade, TLR agonists, MIF inhibition, and STAT3 modulation, as well as nanotechnology-based TAM targeting.

More specifically, hypoxia, cytokines such as G-CSF, TNF-alpha, and IL-6, mitochondrial DNA alterations, and hormonal signals contribute to the maintenance of CSC stemness [125,126]. Key molecular targets have been identified, including HIF-1α, NF-kB, and β-catenin, alongside surface markers like CD44 and CD133, for which specific antibodies have shown efficacy in preclinical studies. The Wnt/β-catenin signaling pathway, crucial for stemness and chemoresistance, is also implicated in endometriosis and cancers such as EOvC, where *CTNNB1* mutations are often present. This pathway may therefore be a promising target and strategy to overcome therapy resistance in both contexts [127]. Efforts have also been made to harness the tumor-killing activity of Cytokine-Induced Killer (CIK) cells, which recognize stemness-associated genes, such as *Oct4* and *ALDH* [128]. In addition, the TME plays a crucial role in maintaining CSC properties and therapy resistance. The Tumor-Associated Macrophages (TAMs) promote invasion, angiogenesis, and metastasis, particularly under hypoxic conditions, by expressing enzymes such as MMP-2, MMP-7, and MMP-9. Targeting TAMs through nanotechnology-based delivery systems or strategies (CSF-1 receptor blockade, TLR agonists—i.e., imiquimod-, tamoxifen, MIF inhibitors, or STAT3 modulation) offers new therapeutic routes [129,130]. Anti-angiogenic agents, such as Bevacizumab, and targeted inhibitors of invasion-related pathways, directly counteract lesion establishment and expansion. These therapies might be particularly relevant in deep infiltrating endometriosis, in which vascularization and extracellular matrix degradation contribute to the acquisition of a greater lesion aggressiveness.

However, it should be noted that, if on one hand all the above approaches have revealed interesting potential beneficial effects in treating endometriosis, on the other hand other implications and important consequences must be considered: (1) No HIF-1α-targeting agent entered in clinical trials for endometriosis because the systemic inhibition of hypoxia signaling raises concerns regarding vascular homeostasis, wound healing, and reproductive physiology. Therefore, HIF-1α inhibition currently serves as a mechanistic and stratification tool, rather than a viable therapeutic strategy for a benign disease [131,132,133]; (2) Concerning NF-κB inhibition, it reduces lesion burden in preclinical models, but systemic NF-κB blockade is clinically untenable due to its essential role in innate immunity and tissue homeostasis. Consequently, NF-κB should be considered as a contextual factor concerning the disease biology and a downstream convergence node and not a direct therapeutic target in endometriosis [59]; (3) Although the Wnt/β-catenin pathway plays an essential role in normal endometrial cycling, implantation, and tissue regeneration, its pharmacologic inhibition is clinically inappropriate in a benign, fertility-relevant disease. Therefore, in endometriotic conditions, aberrant Wnt/β-catenin signaling should be primarily interpreted as a biological indicator of adaptability and stemness-related behavior. Its use can be effective for lesion stratification and mechanistic understanding but not as candidate for therapeutic blockade [121]; (4) Regarding macrophages, since they hold a central role in reproductive and peritoneal homeostasis, their broad depletion is neither justified nor safe in endometriosis. Thus, macrophage signatures are best leveraged as contextual biomarkers of lesion activity, inflammatory burden, and microenvironmental permissiveness [4,133]. Another area of intervention could be represented by highly innovative approaches, including immunotherapy (e.g., checkpoint inhibitors), Cancer Stem Cell (CSC) targeting, nanotechnology-based drug delivery, and gene editing (*CRISPR/Cas9*). These therapeutic strategies would represent the most speculative tier and map the future directions.

The distinctive feature on which these approaches are based is the stemness phenotype demonstrated in ectopic endometrial tissue. Specifically, if therapeutic resistance in endometriosis may be explained by the stemness phenotype itself, this behavior mirrors those observed in malignancies. The presence of stem cell-related features in endometriosis (i.e., expression of pluripotency markers, a stem cell-like subpopulation—ESCs and MenSC-derived progenitors—and activation of pathways such as Wnt/β-catenin and autophagy) contribute to both therapeutic resistance and risk of recurrence. For this reason, targeted CSC-based therapies, including inhibitors of β-catenin, antibodies against CD44/CD133, CIK cell-based strategies, and autophagy modulators, are mechanistically relevant in endometriosis. Also, Menstrual blood-derived Stem Cells (MenSCs) non-invasively obtained exhibit low immunogenicity and tumorigenicity, with high differentiation potential. Studies suggest their superiority over Bone Marrow Mesenchymal Stem Cells (BM-MSCs) in multiple applications [134]. Similarly, fetal stem cells derived from Wharton’s jelly, placenta, or amniotic fluid can be banked for future autologous use, offering low mutation rates and high therapeutic versatility. However, current hormone therapies primarily target differentiated ectopic cells, but they may fail to eliminate the underlying endometrial stem cells responsible for lesion propagation and persistence [135].

As a result, understanding the biology of these cells would be essential for developing lasting and effective therapies. Since endometriosis exhibits resistance to conventional therapies and recurrence, new therapeutic perspectives are focused on targeting CSCs. Autophagy allows these cells to survive under metabolic stress by regenerating ATP. Although all the strategies targeting CSCs in oncology (i.e., Notch, Wnt or autophagy inhibitors) have reported beneficial effects in the oncological field, none of these approaches have been taken forward to the clinical trial stage for endometriosis. Given the fundamental role of stem and progenitor cells in normal endometrial regeneration, implantation and fertility, direct targeting of stem cell pathways carries a high risk of off-target toxicity and reproductive damage; for this reason, these approaches are considered ethically and clinically unsuitable for routine use in a benign disease [103,121,122].

Immunotherapies such as anti-PD1/PD-L1 agents and miRNAs have also been proposed as future tools for diagnosis and treatment [136]. Natural compounds like All-Trans Retinoic Acid (ATRA) and *EZH2* inhibitors (e.g., 3-deazaneplanocin) have demonstrated effects in reversing Epithelial–Mesenchymal Transition (EMT), reducing lesion growth, fibrosis, and inflammation in endometriosis [137,138]. Moreover, Tacrolimus, already in use for female infertility in Polycystic Ovary Syndrome (PCOS), may have therapeutic potential in treating hormone receptor resistance in both endometriosis and cancer [139,140].

Gene-targeting strategies using viral (lentiviral, adenoviral, AAV) or non-viral vectors (e.g., liposomes) are being explored to deliver therapeutic genes to pathological tissues [141]. These delivery methods, including nanoparticles, are being designed to increase the bioavailability and precision of anti-Cancer Stem Cell (anti-CSC) agents, like salinomycin, amlodipine, or metformin. Salinomycin’s efficacy, for instance, was significantly enhanced when conjugated with hyaluronic acid-based nanogels targeting CD44+ drug-resistant cells [142,143].

Thus, these strategies offer the real possibility of a more radical cure by addressing the disease at its genomic or stem cell root; nevertheless, they are currently in early experimental stages and require extensive validation regarding their long-term impact on female fertility and teratogenicity.

Regarding the translational research field, other advanced strategies include endometrial organoids from patients, which reliably reproduce the biological complexity of these lesions [144,145]. Today, these types of approaches constitute a powerful platform for both translational research and the testing of new therapies potentially able to modulate proliferation, inflammation, or survival pathways. The great advantage of using these models lies in comparing the eutopic endometrium with peritoneal lesions derived from ovarian endometriomas, allowing test drug sensitivity on the specific tumor subtype [146,147].

To conclude, very recent approaches involve spatial profiling to detect cellular diversity and the organization of cells in space affected by endometriotic lesions [148,149]. Thus, it is possible to identify stem cell-like populations, inflammatory and hypoxia-sensitive niches, and immune cell states (e.g., pro-angiogenic macrophages) that promote persistence, invasion, and resistance to treatment.

By integrating these two approaches, spatial-omics and organoids, it would be possible to discover new targets, verify whether specific microenvironment-dependent vulnerabilities can be exploited for therapeutic purposes, and identify patient-specific treatment strategies [145].

The complexity of this therapeutic landscape, based on molecular, hormonal, immunologic, and stemness-related processes, reflects the same complexity observed in endometriotic condition. Therefore, if endometriosis is not a malignant condition, its biology justifies exploration of targeted strategies traditionally associated with oncology, and the wide range of therapies may improve long-term disease control, reduce recurrence, and better satisfy the need for mechanism-based treatments. Contextually, it is essential to point out that precisely both the non-malignant nature of endometriosis and the potential adverse effects of almost all the therapeutic approaches in reproductive-age women mean that these in vitro and in vivo therapeutic strategies have not yet entered clinical trials.

Altogether, the translational feasibility of treatments is strictly governed by the benign and chronic nature of the disease. Indeed, unlike oncology, where systemic toxicity is often an accepted trade-off for survival, endometriosis management in women of reproductive age must prioritize the preservation of ovarian function and future fertility [150]. Many of the proposed targeted agents, such as PI3K/mTOR inhibitors and anti-angiogenic compounds, carry significant risks of ovarian toxicity, potentially leading to premature ovarian insufficiency or diminished ovarian reserve [151,152]. Furthermore, given the long-term treatment required for chronic conditions, the teratogenic potential of these small molecules remains a primary concern, as many interfere with fundamental pathways of embryogenesis and vascularization [153]. Therefore, the development of targeted strategies must shift toward locally delivered systems (e.g., nanoparticle-mediated delivery) or therapies with high selectivity for ectopic vs. eutopic tissue, in order to mitigate systemic side effects and safeguard reproductive health.

## 7. Conclusions

Endometriosis, though a benign disease, exhibits some biological behaviors closely resembling those of malignancies, such as cellular invasion, recurrence, and therapeutic resistance [154]. A more in-depth understanding of these features can certainly help in identifying new potential biomarkers for diagnosis and treatment [155]. Indeed, the overlap between endometriosis and certain gynecological cancers, specifically EOvC and OCCC, only wants to underscore the benefits of integrating oncological research strategies into the management of endometriosis. The identification of high-risk patient subgroups, such as those with atypical endometriosis, chronic estrogen stimulation, or genetic mutations in ARID1A and CTNNB1, offers new avenues for targeted surveillance and early intervention. At the same time, advanced experimental models, including stem cell-based systems and 3D cultures, are providing deeper insights into the shared pathophysiological mechanisms between endometriosis and cancer. On the therapeutic front, hormone resistance (progestagens), improper use of estrogens (not only for endometriosis but also for the increased risk of some type of hormonal cancer associated, like melanoma and breast cancer) and disease persistence appear to be strongly associated with the presence of stem-like cells, analogous to CSCs, which are emerging as critical therapeutic targets. The TME of endometriosis lesions, including hypoxia and the role of TAMs, further supports the use of anti-cancer strategies. Novel approaches such as immunotherapies, CSC-targeting agents, nanotechnology-based drug delivery systems, and gene editing techniques offer promising future directions. Therefore, it is important to emphasize that endometriosis is a benign condition with an associated risk of 1.5–2% of developing a neoplastic disease; thus, the common molecular features do not inevitable imply malignant progression. Genetic alterations and the activation of specific pathways would not represent necessarily initial oncogenic events, but rather adaptations to hostile ectopic conditions. Therefore, these molecular traits would constitute selective advantages in a chronically inflamed and estrogen-rich microenvironment, favoring the persistence of lesions, clonal expansion over time and, therefore, also, biological similarity with neoplastic processes, without necessarily implying a deterministic malignant transformation. Thus, a more nuanced understanding of endometriosis as a systemic, stem cell-driven and estrogen-dependent condition may improve both the accuracy of scientific research on the field and clinical management.

## Figures and Tables

**Table 1:** Adenogenetic factors and their cancer-related mechanisms in ovarian tumors.

Marker	Role in Endometriosis	Evidence in Ovarian Cancer	Reference
IGF1/2	Proliferation and survival.	Activation of the IGF-1R receptor stimulates the PI3K/AKT/mTOR signaling pathways, which are frequently mutated (e.g., PIK3CA mutation) in atypical endometriotic lesions. Elevated levels of IGF-1 and IGF-2 in peritoneal and cyst fluid are associated with the progression of endometriosis to OCCC and EOvC.	[59]
PRL-R	Epithelial differentiation.	The prolactin receptor is expressed in both endometriotic lesions and ovarian tumors. Prolactin signaling has been linked to cell cycle progression and the motility of ovarian tumor cells, acting in synergy with oestrogens to enhance the transcription of oncogenic genes.	[60,61,62]
FGF7/10/23	Neo-angiogenesis.	Paracrine factors, secreted by stromal cells, act on the FGFR2b receptors of epithelial cells. In ovarian carcinogenesis, their dysregulation promotes uncontrolled cell proliferation and chemoresistance. Recently identified as a potential biomarker in EAOC, FGF23 not only regulates calcium–phosphate metabolism but also acts as an oncogenic factor via the MAPK pathway, promoting ovarian tumor growth.	[63,64,65]
IFN-τ	Immune evasion.	Dysregulation of these pathways contributes to immune evasion, allowing pre-malignant endometrial cells to escape the control of natural killer (NK) cells and cytotoxic T lymphocytes.	[66,67]
HGF	Invasion and migration.	HGF secreted by macrophages in the endometrial microenvironment creates a tumor-promoting niche that facilitates the survival of cells with ARID1A mutations. The HGF/c-MET axis promotes Epithelial–Mesenchymal Transition (EMT), enabling endometrial cells to invade the ovarian stroma and transform into malignant cells.	[68]

Adenogenetic markers and their role in the biology of endometriosis and ovarian tumors, in particular Endometriosis-Associated Ovarian Carcinomas (EAOCs), such as clear cell carcinoma (OCCC) and endometrioid carcinoma (EOvC). Abbreviations: IGF1: Insulin-like Growth Factor-I; IGF2: Insulin-like Growth Factor-II; PRL-R: PRoLactin Receptor; FGF7: Fibroblast Growth Factor 7; FGF10: Fibroblast Growth Factor 10; FGF23: Fibroblast Growth Factor 23; IFN-τ: Interferon-τ; HGF: Hepatocyte Growth Factor.

**Table 2:** Key genetic and molecular alterations shared between endometriosis and EAOCs.

Gene/ Molecular Target	Molecular Function	Pathway Involved	Cancer Association	Implication in Endometriosis	References
ARID1A	Chromatin remodeling, tumor suppressor	PI3K/AKT/mTOR	OCCC, EOvC	Frequently mutated in ovarian endometriotic lesions; loss promotes malignant transformation	[58,69,73]
PIK3CA	Catalytic subunit of PI3K; oncogenic activator	PI3K/AKT/mTOR	OCCC, EOvC	Activating mutations cooperate with ARID1A loss to drive carcinogenesis	[73,75]
PTEN	Tumor suppressor, inhibits AKT signaling	PI3K/AKT/mTOR	EOvC	Loss of function enhance cell survival and proliferation	[73,75]
KRAS	GTPase, signal transducer	MAPK/RAS–RAF–MEK–ERK	Low-grade serous and endometrioid carcinomas	Activating mutations observed in deep endometriosis; promote proliferation	[73,75]
CTNNB1 (β-catenin)	Transcriptional co-activator	Wnt/β-catenin	EOvC	Stabilization enhances invasion, EMT and stemness	[73,75]
TP53	DNA repair and cell cycle regulator	p53 pathway	High-grade serous carcinomas	Altered expression in atypical or inflamed endometriotic lesions	[73,75]
HNF1B	Transcription factor	Glucose metabolism, differentiation	OCCC	Overexpressed in OCCC and some endometriotic lesions, marker of clear cell lineage	[73,75]

Summary of key genetic and molecular features shared by endometriosis and Endometriosis-Associated Ovarian Cancers (EAOCs). The table details main genes, their role and pathways in which they are involved; these dysregulated cellular pathways may contribute to disease pathogenesis and may promote tumoral forms listed in the table, as well as the progression from benign endometriotic lesions to malignant transformation. Abbreviations: ARID1A: AT-Rich Interactive Domain 1A; PIK3CA: Phosphatidylinositol-4,5-bisphosphate 3-Kinase Catalytic subunit Alpha; PTEN: Phosphatase and Tensin homolog; KRAS: Kirsten Rat Sarcoma Virus; CTNNB1: Catenin Beta 1; TP53: Tumor Protein 53; HNF1B: Hepatocyte Nuclear Factor 1 Beta.

**Table 3:** Hierarchy of evidence and safety for endometriosis treatment.

Level of Evidence	Therapeutic Strategy	Primary Mechanism	Clinical Status/Feasibility	Reproductive Safety Concerns
Standard Care	Hormonal Therapy/Surgery	Estrogen suppression/Physical removal	Gold Standard; high safety but high recurrence.	Minimal risk; reversible effects on ovulation [114]
Preclinical/Repurposed	PI3K/mTOR, PARP Inhibitors, Anti-angiogenics	Targeting proliferation and survival pathways	Experimental (Animal models); concerns about ovarian toxicity.	High risk of ovarian toxicity; potential teratogenicity, interference with oocyte DNA repair mechanisms and risk of impaired corpus luteum formation and subfertility [115]
Early Experimental	Immunotherapy/CSC-targeting	Modulating immune evasion and stemness	Biological Hypothesis; potential for specific targeting.	Effects on maternal–fetal immune tolerance, ovarian inflammation, cross-reactivity with healthy endometrial stem cells and alteration of the ovarian stem cell niche [116]
Future/Speculative	Nanotechnology/Gene Editing	Genomic repair and targeted delivery	Discovery Phase; high translational hurdles regarding reproductive safety	Bioaccumulation of nanoparticles, embryonic toxicity, off-target effects and permanent alterations on the germline and teratogenicity [117,118]

Schematic summary on current therapeutic approaches for endometriosis and evaluation of frontier procedures, considering both their molecular efficacy and reproductive safety and clinical feasibility.

## Data Availability

No new data were created or analyzed in this study. Data sharing is not applicable to this article.

## Abbreviations

The following abbreviations are used in this manuscript:

Anti-CSC	Anti-Cancer Stem Cell
ATRA	All-Trans Retinoic Acid
BM-MSCs	Bone Marrow Mesenchymal Stem Cells
EAOC	Endometriosis-Associated Ovarian Cancer
EMT	Epithelial–Mesenchymal Transition
ESC	Endometriosis Stem Cell
ERON	Endometriosis-Related Ovarian Neoplasm
ERα	Estrogen Receptor α
ERβ	Estrogen Receptor β
DE	Deep Endometriosis
EOvC	Endometrioid Ovarian Carcinoma
GWAS	Genome-Wide Association Studies
MenSCs	Menstrual blood-derived Stem Cells
MMP	Metalloproteinase
OCCC	Clear Cell Ovarian Carcinoma
OE	Ovarian Endometriosis
OC	Ovarian Cancer
PCOS	Polycystic Ovary Syndrome
TME	Tumor Microenvironment
2D	Two-dimensional
3D	Three-dimensional

## References

[1] Guo C., Zhang C. (2024). Role of the gut microbiota in the pathogenesis of endometriosis: A review. Front. Microbiol..

[2] Lamceva J., Uljanovs R., Strumfa I. (2023). The Main Theories on the Pathogenesis of Endometriosis. Int. J. Mol. Sci..

[3] Seli E., Berkkanoglu M., Arici A. (2003). Pathogenesis of endometriosis. Obstet. Gynecol. Clin. N. Am..

[4] Chen S., Liu Y., Zhong Z., Wei C., Liu Y., Zhu X. (2023). Peritoneal immune microenvironment of endometriosis: Role and therapeutic perspectives. Front. Immunol..

[5] Izumi G., Koga K., Takamura M., Makabe T., Satake E., Takeuchi A., Taguchi A., Urata Y., Fujii T., Osuga Y. (2018). Involvement of immune cells in the pathogenesis of endometriosis. J. Obstet. Gynaecol. Res..

[6] Boccellino M., Quagliuolo L., Verde A., La Porta R., Crispi S., Piccolo M.T., Vitiello A., Baldi A., Signorile P.G. (2012). In vitro model of stromal and epithelial immortalized endometriotic cells. J. Cell Biochem..

[7] Keukens A., Veth V.B., Regis M., Mijatovic V., Bongers M.Y., Coppus S.F.P.J., Maas J.W.M. (2024). The effect of surgery or medication on pain and quality of life in women with endometrioma. A systematic review and meta-analysis. Eur. J. Obstet. Gynecol. Reprod. Biol..

[8] Pardanani S., Barbieri R.L. (1998). The gold standard for the surgical diagnosis of endometriosis: Visual findings or biopsy results?. J. Gynecol. Tech..

[9] Liu Z., Gou Y., Zhang H., Zuo H., Zhang H., Liu Z., Yao D. (2014). Estradiol improves cardiovascular function through up-regulation of SOD2 on vascular wall. Redox Biol..

[10] Klinge C.M. (2020). Estrogenic control of mitochondrial function. Redox Biol..

[11] Su E.J., Lin Z.H., Zeine R., Yin P., Reierstad S., Innes J.E., Bulun S.E. (2009). Estrogen receptor-beta mediates cyclooxygenase-2 expression and vascular prostanoid levels in human placental villous endothelial cells. Am. J. Obstet. Gynecol..

[12] Hudelist G., Keckstein J., Czerwenka K., Lass H., Walter I., Auer M., Wieser F., Wenzl R., Kubista E., Singer C.F. (2005). Estrogen receptor beta and matrix metalloproteinase 1 are coexpressed in uterine endometrium and endometriotic lesions of patients with endometriosis. Fertil. Steril..

[13] Kvaskoff M., Mu F., Terry K.L., Harris H.R., Poole E.M., Farland L., Missmer S.A. (2015). Endometriosis: A high-risk population for major chronic diseases?. Hum. Reprod. Update.

[14] Shafrir A.L., Farland L.V., Shah D.K., Harris H.R., Kvaskoff M., Zondervan K., Missmer S.A. (2018). Risk for and consequences of endometriosis: A critical epidemiologic review. Best Pract. Res. Clin. Obstet. Gynaecol..

[15] Bogani G., Chiappa V., Raspagliesi F., Corso G. (2025). Endometriosis and cancer risk. Eur. J. Cancer Prev..

[16] Li J., Liu R., Tang S., Feng F., Liu C., Wang L., Zhao W., Zhang T., Yao Y., Wang X. (2019). Impact of endometriosis on risk of ovarian, endometrial and cervical cancers: A meta-analysis. Arch. Gynecol. Obstet..

[17] Cousins F.L., Dorien F.O., Gargett C.E. (2018). Endometrial stem/progenitor cells and their role in the pathogenesis of endometriosis. Best Pract. Res. Clin. Obstet. Gynaecol..

[18] Smolarz B., Szyłło K., Romanowicz H. (2021). Endometriosis: Epidemiology, Classification, Pathogenesis, Treatment and Genetics (Review of Literature). Int. J. Mol. Sci..

[19] Bulun S.E. (2009). Endometriosis. N. Engl. J. Med..

[20] Bulun S.E., Yilmaz B.D., Sison C., Miyazaki K., Bernardi L., Liu S., Kohlmeier A., Yin P., Milad M., Wei J. (2019). Endometriosis. Endocr. Rev..

[21] Bulun S.E., Monsavais D., Pavone M.E., Dyson M., Xue Q., Attar E., Tokunaga H., Su E.J. (2012). Role of estrogen receptor-β in endometriosis. Semin. Reprod. Med..

[22] Monsivais D., Dyson M.T., Yin P., Coon J.S., Navarro A., Feng G., Malpani S.S., Ono M., Ercan C.M., Wei J.J. (2014). ERβ- and prostaglandin E2-regulated pathways integrate cell proliferation via Ras-like and estrogen-regulated growth inhibitor in endometriosis. Mol. Endocrinol..

[23] Pavone M.E., Bulun S.E. (2012). Aromatase inhibitors for the treatment of endometriosis. Fertil. Steril..

[24] Taylor H.S., Kotlyar A.M., Flores V.A. (2021). Endometriosis is a chronic systemic disease: Clinical challenges and novel innovations. Lancet.

[25] Zondervan K.T., Becker C.M., Koga K., Missmer S.A., Taylor R.N., Viganò P. (2018). Endometriosis. Nat. Rev. Dis. Primers.

[26] Xue Q., Lin Z., Cheng Y.H., Huang C.C., Marsh E., Yin P., Milad M.P., Confino E., Reierstad S., Innes J. (2007). Promoter methylation regulates estrogen receptor 2 in human endometrium and endometriosis. Biol. Reprod..

[27] Patel B.G., Rudnicki M., Yu J., Shu Y., Taylor R.N. (2017). Progesterone resistance in endometriosis: Origins, consequences and interventions. Acta Obstet. Gynecol. Scand..

[28] Zondervan K.T., Becker C.M., Missmer S.A. (2020). Endometriosis. N. Engl. J. Med..

[29] Saunders P.T.K., Horne A.W. (2021). Endometriosis: Etiology, pathobiology, and therapeutic prospects. Cell.

[30] González-Ramos R., Defrère S., Devoto L. (2012). Nuclear factor-kappaB: A main regulator of inflammation and cell survival in endometriosis pathophysiology. Fertil. Steril..

[31] Ni C., Li D. (2024). Ferroptosis and oxidative stress in endometriosis: A systematic review of the literature. Medicine.

[32] Huang L., Shi L., Li M., Yin X., Ji X. (2025). Oxidative stress in endometriosis: Sources, mechanisms and therapeutic potential of antioxidants (Review). Int. J. Mol. Med..

[33] Cuffaro F., Russo E., Amedei A. (2024). Endometriosis, Pain, and Related Psychological Disorders: Unveiling the Interplay among the Microbiome, Inflammation, and Oxidative Stress as a Common Thread. Int. J. Mol. Sci..

[34] Scutiero G., Iannone P., Bernardi G., Bonaccorsi G., Spadaro S., Volta C.A., Greco P., Nappi L. (2017). Oxidative Stress and Endometriosis: A Systematic Review of the Literature. Oxidative Med. Cell. Longev..

[35] Carvalho L.F., Samadder A.N., Agarwal A., Fernandes L.F., Abrão M.S. (2012). Oxidative stress biomarkers in patients with endometriosis: Systematic review. Arch. Gynecol. Obstet..

[36] Van Langendonckt A., Casanas-Roux F., Donnez J. (2002). Iron overload in the peritoneal cavity of women with pelvic endometriosis. Fertil. Steril..

[37] Defrère S., González-Ramos R., Lousse J.C., Colette S., Donnez O., Donnez J., Van Langendonckt A. (2011). Insights into iron and nuclear factor-kappa B (NF-kappaB) involvement in chronic inflammatory processes in peritoneal endometriosis. Histol. Histopathol..

[38] González-Ramos R., Van Langendonckt A., Defrère S., Lousse J.C., Colette S., Devoto L., Donnez J. (2010). Involvement of the nuclear factor-κB pathway in the pathogenesis of endometriosis. Fertil. Steril..

[39] Guo S.W. (2009). Epigenetics of endometriosis. Mol. Hum. Reprod..

[40] Yamagata Y., Nishino K., Takaki E., Sato S., Maekawa R., Nakai A., Sugino N. (2014). Genome-wide DNA methylation profiling in cultured eutopic and ectopic endometrial stromal cells. PLoS ONE.

[41] Li S., Fan Y., Shu C., Zhou Y., Shu J. (2024). Methyl 3,4-dihydroxybenzoate alleviates oxidative damage in granulosa cells by activating Nrf2 antioxidant pathway. J. Ovarian Res..

[42] Cheng X., Memon M.A., Ali W., Ma Y. (2025). Ferroptosis: A novel pathway in the pathogenesis and treatment of endometriosis. J. Mol. Histol..

[43] Zhang T., De Carolis C., Man G.C.W., Wang C.C. (2018). The link between immunity, autoimmunity and endometriosis: A literature update. Autoimmun. Rev..

[44] Chen L.H., Lo W.C., Huang H.Y., Wu H.M. (2023). A Lifelong Impact on Endometriosis: Pathophysiology and Pharmacological Treatment. Int. J. Mol. Sci..

[45] Burney R.O., Giudice L.C. (2012). Pathogenesis and pathophysiology of endometriosis. Fertil. Steril..

[46] Chantalat E., Valera M.C., Vaysse C., Noirrit E., Rusidze M., Weyl A., Vergriete K., Buscail E., Lluel P., Fontaine C. (2020). Estrogen Receptors and Endometriosis. Int. J. Mol. Sci..

[47] Becker C.M., D’Amato R.J. (2007). Angiogenesis and antiangiogenic therapy in endometriosis. Microvasc. Res..

[48] Zhang W., Li K., Jian A., Zhang G., Zhang X. (2025). Prospects for potential therapy targeting immune-associated factors in endometriosis (Review). Mol. Med. Rep..

[49] Symons L.K., Miller J.E., Kay V.R., Marks R.M., Liblik K., Koti M., Tayade C. (2018). The Immunopathophysiology of Endometriosis. Trends Mol. Med..

[50] Wang X., Wu N., Xue Q. (2025). Macrophages in endometriosis: Key roles and emerging therapeutic opportunities-a narrative review. Reprod. Biol. Endocrinol..

[51] García-Gómez E., Vázquez-Martínez E.R., Reyes-Mayoral C., Cruz-Orozco O.P., Camacho-Arroyo I., Cerbón M. (2020). Regulation of Inflammation Pathways and Inflammasome by Sex Steroid Hormones in Endometriosis. Front. Endocrinol..

[52] Kim B.S., Kim B., Yoon S., Park W., Bae S.J., Joo J., Kim W., Ha K.T. (2025). Warburg-like Metabolic Reprogramming in Endometriosis: From Molecular Mechanisms to Therapeutic Approaches. Pharmaceuticals.

[53] Anchan M.M., Dutta R. (2025). Reframing Endometriosis: Interplay of NETs, Macrophages, and Lymphocytes at the Crossroads of Disease Progression, Infertility, and Malignant Transformation. Am. J. Reprod. Immunol..

[54] Jeong J.W., Kwak I., Lee K.Y., Kim T.H., Large M.J., Stewart C.L., Kaestner K.H., Lydon J.P., DeMayo F.J. (2010). Foxa2 is essential for mouse endometrial gland development and fertility. Biol. Reprod..

[55] Franco H.L., Dai D., Lee K.Y., Rubel C.A., Roop D., Boerboom D., Jeong J.W., Lydon J.P., Bagchi I.C., Bagchi M.K. (2011). WNT4 is a key regulator of normal postnatal uterine development and progesterone signaling during embryo implantation and decidualization in the mouse. FASEB J..

[56] Spencer T.E., Dunlap K.A., Filant J. (2012). Comparative developmental biology of the uterus: Insights into mechanisms and developmental disruption. Mol. Cell. Endocrinol..

[57] Signorile P.G., Baldi A., Viceconte R., Boccellino M. (2025). The Role of Adenogenesis Factors in the Pathogenesis of Endometriosis. Int. J. Mol. Sci..

[58] Signorile P.G., Baldi A., Viceconte R., Carraturo E., Boccellino M., Fordellone M., Montella M. (2025). Glycosaminoglycan Adenogenesis Factors: Immunohistochemical Expression in Endometriosis Tissues Compared with the Endometrium. Crit. Rev. Eukaryot. Gene Expr..

[59] Driva T.S., Schatz C., Haybaeck J. (2023). Endometriosis-Associated Ovarian Carcinomas: How PI3K/AKT/mTOR Pathway Affects Their Pathogenesis. Biomolecules.

[60] Ramírez-de-Arellano A., Villegas-Pineda J.C., Hernández-Silva C.D., Pereira-Suárez A.L. (2021). The Relevant Participation of Prolactin in the Genesis and Progression of Gynecological Cancers. Front. Endocrinol..

[61] Levina V.V., Nolen B., Su Y., Godwin A.K., Fishman D., Liu J., Mor G., Maxwell L.G., Herberman R.B., Szczepanski M.J. (2009). Biological significance of prolactin in gynecologic cancers. Cancer Res..

[62] Auriemma R.S., Del Vecchio G., Scairati R., Pirchio R., Liccardi A., Verde N., de Angelis C., Menafra D., Pivonello C., Conforti A. (2020). The Interplay Between Prolactin and Reproductive System: Focus on Uterine Pathophysiology. Front. Endocrinol..

[63] Feng S., Ding B., Dai Z., Yin H., Ding Y., Liu S., Zhang K., Lin H., Xiao Z., Shen Y. (2024). Cancer-associated fibroblast-secreted FGF7 as an ovarian cancer progression promoter. J. Transl. Med..

[64] Edirisinghe O., Ternier G., Kumar T.K.S. (2025). Pathology and Therapeutic Significance of Fibroblast Growth Factors. Targets.

[65] Tebben P.J., Kalli K.R., Cliby W.A., Hartmann L.C., Grande J.P., Singh R.J., Kumar R. (2005). Elevated fibroblast growth factor 23 in women with malignant ovarian tumors. Mayo Clin. Proc..

[66] Peng Y., Zhong G., Zhou M., Yao Y., Dong K., Yang Z., An L., Zhang J., Zhang J., Zhang S. (2026). The dysregulation of the immune microenvironment during endometrial intraepithelial neoplasia serves as a marker of endometrial carcinogenesis. Front. Immunol..

[67] Jiang W., Xu W., Chen F. (2025). Dysfunction of natural killer cells promotes immune escape and disease progression in endometriosis. Front. Immunol..

[68] Owusu B.Y., Galemmo R., Janetka J., Klampfer L. (2017). Hepatocyte Growth Factor, a Key Tumor-Promoting Factor in the Tumor Microenvironment. Cancers.

[69] Sapkota Y., Steinthorsdottir V., Morris A.P., Fassbender A., Rahmioglu N., De Vivo I., Buring J.E., Zhang F., Edwards T.L., Jones S. (2017). Meta-analysis identifies five novel loci associated with endometriosis highlighting key genes involved in hormone metabolism. Nat. Commun..

[70] Nyholt D.R., Low S.K., Anderson C.A., Painter J.N., Uno S., Morris A.P., MacGregor S., Gordon S.D., Henders A.K., Martin N.G. (2012). Genome-wide association meta-analysis identifies new endometriosis risk loci. Nat. Genet..

[71] Lappalainen T., Sammeth M., Friedländer M.R., ‘t Hoen P.A., Monlong J., Rivas M.A., Gonzàlez-Porta M., Kurbatova N., Griebel T., Ferreira P.G. (2013). Transcriptome and genome sequencing uncovers functional variation in humans. Nature.

[72] Nakaoka H., Gurumurthy A., Hayano T., Ahmadloo S., Omer W.H., Yoshihara K., Yamamoto A., Kurose K., Enomoto T., Akira S. (2016). Allelic Imbalance in Regulation of ANRIL through Chromatin Interaction at 9p21 Endometriosis Risk Locus. PLoS Genet..

[73] Anglesio M.S., Bashashati A., Wang Y.K., Senz J., Ha G., Yang W., Aniba M.R., Prentice L.M., Farahani H., Li Chang H. (2015). Multifocal endometriotic lesions associated with cancer are clonal and carry a high mutation burden. J. Pathol..

[74] Er T.K., Su Y.F., Wu C.C., Chen C.C., Wang J., Hsieh T.H., Herreros-Villanueva M., Chen W.T., Chen Y.T., Liu T.C. (2016). Targeted next-generation sequencing for molecular diagnosis of endometriosis-associated ovarian cancer. J. Mol. Med..

[75] Argentati C., Tortorella I., Bazzucchi M., Morena F., Martino S. (2020). Harnessing the Potential of Stem Cells for Disease Modeling: Progress and Promises. J. Pers. Med..

[76] Caumanns J.J., Wisman G.B.A., Berns K., van der Zee A.G.J., de Jong S. (2018). ARID1A mutant ovarian clear cell carcinoma: A clear target for synthetic lethal strategies. Biochim. Biophys. Acta Rev. Cancer.

[77] Berns K., Caumanns J.J., Hijmans E.M., Gennissen A.M.C., Severson T.M., Evers B., Wisman G.B.A., Jan Meersma G., Lieftink C., Beijersbergen R.L. (2018). ARID1A mutation sensitizes most ovarian clear cell carcinomas to BET inhibitors. Oncogene.

[78] Kuo K.T., Mao T.L., Jones S., Veras E., Ayhan A., Wang T.L., Glas R., Slamon D., Velculescu V.E., Kuman R.J. (2009). Frequent activating mutations of PIK3CA in ovarian clear cell carcinoma. Am. J. Pathol..

[79] Tong A., Di X., Zhao X., Liang X. (2023). Review the progression of ovarian clear cell carcinoma from the perspective of genomics and epigenomics. Front. Genet..

[80] Hollis R.L., Thomson J.P., Stanley B., Churchman M., Meynert A.M., Rye T., Bartos C., Iida Y., Croy I., Mackean M. (2020). Molecular stratification of endometrioid ovarian carcinoma predicts clinical outcome. Nat. Commun..

[81] Martins F.C., Couturier D.L., Paterson A., Karnezis A.N., Chow C., Nazeran T.M., Odunsi A., Gentry-Maharaj A., Vrvilo A., Hein A. (2020). Clinical and pathological associations of PTEN expression in ovarian cancer: A multicentre study from the Ovarian Tumour Tissue Analysis Consortium. Br. J. Cancer.

[82] Orr N.L., Albert A., Liu Y.D., Lum A., Hong J., Ionescu C.L., Senz J., Nazeran T.M., Lee A.F., Noga H. (2023). KRAS mutations and endometriosis burden of disease. J. Pathol. Clin. Res..

[83] Pejovic T., Cathcart A.M., Alwaqfi R., Brooks M.N., Kelsall R., Nezhat F.R. (2024). Genetic Links between Endometriosis and Endometriosis-Associated Ovarian Cancer-A Narrative Review (Endometriosis-Associated Cancer). Life.

[84] Wiegand K.C., Shah S.P., Al-Agha O.M., Zhao Y., Tse K., Zeng T., Senz J., McConechy M.K., Anglesio M.S., Kalloger S.E. (2010). ARID1A mutations in endometriosis-associated ovarian carcinomas. N. Engl. J. Med..

[85] Vitale I., Manic G., De Maria R., Kroemer G., Galluzzi L. (2017). DNA Damage in Stem Cells. Mol. Cell..

[86] Hsieh C.J., Klump B., Holzmann K., Borchard F., Gregor M., Porschen R. (1998). Hypermethylation of the p16INK4a promoter in colectomy specimens of patients with long-standing and extensive ulcerative colitis. Cancer Res..

[87] Issa J.P., Ahuja N., Toyota M., Bronner M.P., Brentnall T.A. (2001). Accelerated age-related CpG island methylation in ulcerative colitis. Cancer Res..

[88] Yin X., Pavone M.E., Lu Z., Wei J., Kim J.J. (2012). Increased activation of the PI3K/AKT pathway compromises decidualization of stromal cells from endometriosis. J. Clin. Endocrinol. Metab..

[89] Zhang H., Li M., Zheng X., Sun Y., Wen Z., Zhao X. (2009). Endometriotic stromal cells lose the ability to regulate cell-survival signaling in endometrial epithelial cells in vitro. Mol. Hum. Reprod..

[90] Bernardi L.A., Dyson M.T., Tokunaga H., Sison C., Oral M., Robins J.C., Bulun S.E. (2019). The Essential Role of GATA6 in the Activation of Estrogen Synthesis in Endometriosis. Reprod. Sci..

[91] McKinnon B.D., Kocbek V., Nirgianakis K., Bersinger N.A., Mueller M.D. (2016). Kinase signalling pathways in endometriosis: Potential targets for non-hormonal therapeutics. Hum. Reprod. Update.

[92] Stewart K.A., Cope A.G., Burnett T.L., Green I.C. (2025). Deep endometriosis demystified: Natural progression, hormonal treatment, and malignant transformation. Curr. Opin. Obstet. Gynecol..

[93] Kvaskoff M., Mahamat-Saleh Y., Farland L.V., Shigesi N., Terry K.L., Harris H.R., Roman H., Becker C.M., As-Sanie S., Zondervan K.T. (2021). Endometriosis and cancer: A systematic review and meta-analysis. Hum. Reprod. Update.

[94] Sorbi F., Capezzuoli T., Saso S., Fambrini M., Corda M., Fantappiè G., Petraglia F. (2021). The relation between endometrioma and ovarian cancer. Minerva Obstet. Gynecol..

[95] Prat J. (2012). Ovarian carcinomas: Five distinct diseases with different origins, genetic alterations, and clinicopathological features. Virchows Arch..

[96] Bas-Esteve E., Pérez-Arguedas M., Guarda-Muratori G.A., Acién M., Acién P. (2019). Endometriosis and ovarian cancer: Their association and relationship. Eur. J. Obstet. Gynecol. Reprod. Biol. X.

[97] Maeda D., Shih I.e.M. (2013). Pathogenesis and the role of ARID1A mutation in endometriosis-related ovarian neoplasms. Adv. Anat. Pathol..

[98] Chen B., Zhao L., Yang R., Xu T. (2024). New insights about endometriosis-associated ovarian cancer: Pathogenesis, risk factors, prediction and diagnosis and treatment. Front. Oncol..

[99] Yamaguchi K., Mandai M., Toyokuni S., Hamanishi J., Higuchi T., Takakura K., Fujii S. (2008). Contents of endometriotic cysts, especially the high concentration of free iron, are a possible cause of carcinogenesis in the cysts through the iron-induced persistent oxidative stress. Clin. Cancer Res..

[100] Wei J.J., William J., Bulun S. (2011). Endometriosis and ovarian cancer: A review of clinical, pathologic, and molecular aspects. Int. J. Gynecol. Pathol..

[101] Akbarzadeh-Jahromi M., Shekarkhar G., Sari Aslani F., Azarpira N., Heidari Esfahani M., Momtahan M. (2015). Prevalence of Endometriosis in Malignant Epithelial Ovarian Tumor. Arch. Iran. Med..

[102] Barnard M.E., Farland L.V., Yan B., Wang J., Trabert B., Doherty J.A., Meeks H.D., Madsen M., Guinto E., Collin L.J. (2024). Endometriosis Typology and Ovarian Cancer Risk. JAMA.

[103] Wilczyński J.R., Szubert M., Paradowska E., Wilczyński M. (2022). Endometriosis Stem Cells as a Possible Main Target for Carcinogenesis of Endometriosis-Associated Ovarian Cancer (EAOC). Cancers.

[104] Song Y., Xiao L., Fu J., Huang W., Wang Q., Zhang X., Yang S. (2014). Increased expression of the pluripotency markers sex-determining region Y-box 2 and Nanog homeobox in ovarian endometriosis. Reprod. Biol. Endocrinol..

[105] Di Claudio K.P. (2016). Endometriosis: Involvement of Stem Cells and Clinical Impact. Ph.D. Thesis.

[106] Signorile P.G., Baldi F., Bussani R., D’Armiento M., De Falco M., Boccellino M., Quagliuolo L., Baldi A. (2010). New evidence of the presence of endometriosis in the human fetus. Reprod. Biomed. Online.

[107] Gargett C.E., Schwab K.E., Deane J.A. (2016). Endometrial stem/progenitor cells: The first 10 years. Hum. Reprod. Update.

[108] Laudański P., Rogalska G., Warzecha D., Lipa M., Mańka G., Kiecka M., Spaczyński R., Piekarski P., Banaszewska B., Jakimiuk A. (2023). Autoantibody screening of plasma and peritoneal fluid of patients with endometriosis. Hum. Reprod..

[109] Filby C.E., Rombauts L., Montgomery G.W., Giudice L.C., Gargett C.E. (2020). Cellular Origins of Endometriosis: Towards Novel Diagnostics and Therapeutics. Semin. Reprod. Med..

[110] Han S.J., Lee J.E., Cho Y.J., Park M.J., O’Malley B.W. (2019). Genomic Function of Estrogen Receptor β in Endometriosis. Endocrinology.

[111] Yuan M., Chen S., Liao Z., Wang K. (2025). The expression of autophagy-related gene CXCL12 in endometriosis associated ovarian cancer and pan-cancer analysis. Front. Endocrinol..

[112] Ravegnini G., Coadă C.A., Mantovani G., De Leo A., de Biase D., Costantino A., Gorini F., Dondi G., Di Costanzo S., Mezzapesa F. (2025). MicroRNA profiling reveals potential biomarkers for the early transformation of endometriosis towards endometriosis-correlated ovarian cancer. Transl. Oncol..

[113] Sohel H.I., Kiyono T., Zahan U.F., Razia S., Ishikawa M., Yamashita H., Kanno K., Sonia S.B., Nakayama K., Kyo S. (2025). Establishment of a Novel In Vitro and In Vivo Model to Understand Molecular Carcinogenesis of Endometriosis-Related Ovarian Neoplasms. Int. J. Mol. Sci..

[114] Vannuccini S., Clemenza S., Rossi M., Petraglia F. (2022). Hormonal treatments for endometriosis: The endocrine background. Rev. Endocr. Metab. Disord..

[115] Zheng W., Cao L., Xu Z., Ma Y., Liang X. (2018). Anti-Angiogenic Alternative and Complementary Medicines for the Treatment of Endometriosis: A Review of Potential Molecular Mechanisms. Evid. Based Complement. Altern. Med..

[116] Stefanoudakis D., Karopoulou E., Matsas A., Katsampoula G.A., Tsarna E., Stamoula E., Christopoulos P. (2024). Immunotherapy in Cervical and Endometrial Cancer: Current Landscape and Future Directions. Life.

[117] Lulseged B.A., Ramaiyer M.S., Michel R., Saad E.E., Ozpolat B., Borahay M.A. (2024). The Role of Nanomedicine in Benign Gynecologic Disorders. Molecules.

[118] Jiang Y., Sun R., Fan M., Sheng M. (2023). Applications of nanomaterials in endometriosis treatment. Front. Bioeng. Biotechnol..

[119] Hruda M., Robová H., Sehnal B., Babková A., Pichlík T., Drozenová J., Malíková H., Halaška M.J., Rob L. (2024). Malignant transformation of extragenital endometriosis. Ceska Gynekol..

[120] Mitranovici M.I., Chiorean D.M., Moraru L., Moraru R., Caravia L., Tiron A.T., Cotoi T.C., Toru H.S., Cotoi O.S. (2024). Shared Pathogenic and Therapeutic Characteristics of Endometriosis, Adenomyosis, and Endometrial Cancer: A Comprehensive Literature Review. Pharmaceuticals.

[121] Guo S.W., Groothuis P.G. (2018). Is it time for a paradigm shift in drug research and development in endometriosis/adenomyosis?. Hum. Reprod. Update.

[122] Steinbuch S.C., Lüß A.M., Eltrop S., Götte M., Kiesel L. (2024). Endometriosis-Associated Ovarian Cancer: From Molecular Pathologies to Clinical Relevance. Int. J. Mol. Sci..

[123] Guo S.W. (2020). Cancer-associated mutations in endometriosis: Shedding light on the pathogenesis and pathophysiology. Hum. Reprod. Update.

[124] Laschke M.W., Menger M.D. (2018). Basic mechanisms of vascularization in endometriosis and their clinical implications. Hum. Reprod. Update.

[125] Becker C.M., Bokor A., Heikinheimo O., Horne A., Jansen F., Kiesel L., King K., Kvaskoff M., Nap A., Petersen K. (2022). ESHRE guideline: Endometriosis. Hum. Reprod. Open.

[126] Du J., Zhu X., Guo R., Xu Z., Cheng F.F., Liu Q., Yang F., Guan L., Liu Y., Lin J. (2018). Autophagy induces G0/G1 arrest and apoptosis in menstrual blood-derived endometrial stem cells via GSK3-β/β-catenin pathway. Stem Cell Res. Ther..

[127] Sciacovelli M., Frezza C. (2017). Metabolic reprogramming and epithelial-to-mesenchymal transition in cancer. FEBS J..

[128] Li C., Yang X., Cheng Y., Wang J. (2024). LGR5, a prognostic stem cell target, promotes endometrial cancer proliferation through autophagy activation. Transl. Oncol..

[129] Gammaitoni L., Giraudo L., Leuci V., Todorovic M., Mesiano G., Picciotto F., Pisacane A., Zaccagna A., Volpe M.G., Gallo S. (2013). Effective activity of cytokine-induced killer cells against autologous metastatic melanoma including cells with stemness features. Clin. Cancer Res..

[130] Kang X., Huang Y., Wang H., Jadhav S., Yue Z., Tiwari A.K., Babu R.J. (2023). Tumor-Associated Macrophage Targeting of Nanomedicines in Cancer Therapy. Pharmaceutics.

[131] Eid R.A., Alaa Edeen M., Shedid E.M., Kamal A.S.S., Warda M.M., Mamdouh F., Khedr S.A., Soltan M.A., Jeon H.W., Zaki M.S.A. (2023). Targeting Cancer Stem Cells as the Key Driver of Carcinogenesis and Therapeutic Resistance. Int. J. Mol. Sci..

[132] Wu M.H., Hsiao K.Y., Tsai S.J. (2019). Hypoxia: The force of endometriosis. J. Obstet. Gynaecol. Res..

[133] Garcia-Alonso L., Handfield L.F., Roberts K., Nikolakopoulou K., Fernando R.C., Gardner L., Woodhams B., Arutyunyan A., Polanski K., Hoo R. (2021). Mapping the temporal and spatial dynamics of the human endometrium in vivo and in vitro. Nat. Genet..

[134] Hogg C., Horne A.W., Greaves E. (2020). Endometriosis-Associated Macrophages: Origin, Phenotype, and Function. Front. Endocrinol..

[135] Lv H., Hu Y., Cui Z., Jia H. (2018). Human menstrual blood: A renewable and sustainable source of stem cells for regenerative medicine. Stem Cell Res. Ther..

[136] Huang T., Song X., Xu D., Tiek D., Goenka A., Wu B., Sastry N., Hu B., Cheng S.Y. (2020). Stem cell programs in cancer initiation, progression, and therapy resistance. Theranostics.

[137] Ronsini C., Fumiento P., Iavarone I., Greco P.F., Cobellis L., De Franciscis P. (2023). Liquid Biopsy in Endometriosis: A Systematic Review. Int. J. Mol. Sci..

[138] Torres-Reverón A., Palermo K., Hernández-López A., Hernández S., Cruz M.L., Thompson K.J., Flores I., Appleyard C.B. (2016). Endometriosis Is Associated With a Shift in MU Opioid and NMDA Receptor Expression in the Brain Periaqueductal Gray. Reprod. Sci..

[139] Zhang Q., Dong P., Liu X., Sakuragi N., Guo S.W. (2017). Enhancer of Zeste homolog 2 (EZH2) induces epithelial-mesenchymal transition in endometriosis. Sci. Rep..

[140] Albaghdadi A.J.H., Kan F.W.K. (2021). Therapeutic Potentials of Low-Dose Tacrolimus for Aberrant Endometrial Features in Polycystic Ovary Syndrome. Int. J. Mol. Sci..

[141] Yang H.L., Zhou W.J., Gu C.J., Meng Y.H., Shao J., Li D.J., Li M.Q. (2018). Pleiotropic roles of melatonin in endometriosis, recurrent spontaneous abortion, and polycystic ovary syndrome. Am. J. Reprod. Immunol..

[142] Rosner M., Horer S., Feichtinger M., Hengstschläger M. (2023). Multipotent fetal stem cells in reproductive biology research. Stem Cell Res. Ther..

[143] Silvestris E., Minoia C., Guarini A., Opinto G., Negri A., Dellino M., Tinelli R., Cormio G., Paradiso A.V., De Palma G. (2022). Ovarian Stem Cells (OSCs) from the Cryopreserved Ovarian Cortex: A Potential for Neo-Oogenesis in Women with Cancer-Treatment Related Infertility: A Case Report and a Review of Literature. Curr. Issues Mol. Biol..

[144] Liu Y., Liu Z., Tang H., Shen Y., Gong Z., Xie N., Zhang X., Wang W., Kong W., Zhou Y. (2019). The N6-methyladenosine (m6A)-forming enzyme METTL3 facilitates M1 macrophage polarization through the methylation of STAT1 mRNA. Am. J. Physiol. Cell Physiol..

[145] Ciprietti M., Bueds C., Vankelecom H., Vriens J. (2025). Organoids as powerful models of endometrium epithelium in transcriptomic, cellular and functional mimicry. Cell Mol. Life Sci..

[146] Marr E.E., Gnecco J.S., Missmer S.A., Hawkins S.M., Osteen K.G., Hummelshoj L., Greaves E., Bruner-Tran K.L., EPHect Experimental Models Working Group (2025). WERF Endometriosis Phenome and Biobanking Harmonisation Project for Experimental Models in Endometriosis Research (EPHect-EM-Organoids): Endometrial organoids as an emerging technology for endometriosis research. Mol. Hum. Reprod..

[147] Gunther K., Liu D., Cortesi M., Powell E., Nesbitt-Hawes E., Abbott J.A., Ford C.E. (2026). Patient-derived epithelial cell organoids mimic the phenotypic complexity of endometriosis subtypes. Hum. Reprod..

[148] Zhang R., Yang Y., Li R., Ma Y., Ma S., Chen X., Li B., Li B., Qi X., Ha C. (2025). Construction organoid model of ovarian endometriosis and the function of estrogen and progesterone in the model. Sci. Rep..

[149] Liu S., Li X., Gu Z., Wu J., Jia S., Shi J., Dai Y., Wu Y., Yan H., Zhang J. (2025). Single-cell and spatial transcriptomic profiling revealed niche interactions sustaining growth of endometriotic lesions. Cell Genom..

[150] Rangi S., Hur C., Richards E., Falcone T. (2023). Fertility Preservation in Women with Endometriosis. J. Clin. Med..

[151] Rosario R., Cui W., Anderson R.A. (2022). Potential ovarian toxicity and infertility risk following targeted anti-cancer therapies. Reprod. Fertil..

[152] Zhu Q., Li Y., Ma J., Ma H., Liang X. (2023). Potential factors result in diminished ovarian reserve: A comprehensive review. J. Ovarian Res..

[153] Sadłocha M., Toczek J., Major K., Staniczek J., Stojko R. (2024). Endometriosis: Molecular Pathophysiology and Recent Treatment Strategies-Comprehensive Literature Review. Pharmaceuticals.

[154] Qi Y., Chen X., Zheng S., Wu T., Li Z., Cheng J., Yang X., Tao W., Huang Q., Gu J. (2025). Single-cell and spatially resolved omics reveal transcriptional and metabolic signatures of ovarian endometriomas. Nat. Commun..

[155] Signorile P.G., Cassano M., Viceconte R., Spyrou M., Marcattilj V., Baldi A. (2022). Endometriosis: A Retrospective Analysis on Diagnostic Data in a Cohort of 4401 Patients. In Vivo.

